# Seamlessly fused digital-analogue reconfigurable computing using memristors

**DOI:** 10.1038/s41467-018-04624-8

**Published:** 2018-06-04

**Authors:** Alexantrou Serb, Ali Khiat, Themistoklis Prodromakis

**Affiliations:** Electronic Materials & Devices, Zepler Institute, University of Southampton, Highfield campus, Southampton, SO17 1BJ UK

## Abstract

As the world enters the age of ubiquitous computing, the need for reconfigurable hardware operating close to the fundamental limits of energy consumption becomes increasingly pressing. Simultaneously, scaling-driven performance improvements within the framework of traditional analogue and digital design become progressively more restricted by fundamental physical constraints. Emerging nanoelectronics technologies bring forth new prospects yet a significant rethink of electronics design is required for realising their full potential. Here we lay the foundations of a design approach that fuses analogue and digital thinking by combining digital electronics with analogue memristive devices for achieving charge-based computation; information processing where every dissipated charge counts. This is realised by introducing memristive devices into standard logic gates, thus rendering them reconfigurable and capable of performing analogue computation at a power cost close to digital. The versatility and benefits of our approach are experimentally showcased through a hardware data clusterer and an analogue NAND gate.

## Abstract (web summary)

Conventional analogue and digital circuits are mixed at signal level yet remain technologically separate, adding complexity and cost. This work introduces a new design paradigm where the analogue and digital worlds are seamlessly fused via memristors, enabling electronics with reconfigurability.

## Introduction

Realising the rapid expansion of the Internet of Things (IoT) relies on the availability of energy-, area- and computationally efficient yet affordable and often-reconfigurable hardware platforms that could allow for bespoke customisation^1^. Within the fully digital design paradigm that still dominates modern electronics, downscaling of integrated circuits^2^ has been the main driver for lowering power dissipation; a process now reaching its physical limits. Simultaneously, reconfigurability has continued to rely for its physical implementation on dedicated memory blocks and progressively bottlenecked by power-hungry data transfers between physically separate memory and processing elements^3^. This has occurred despite work on field programmable analogue arrays (FPAAs) based on floating gate MOSFETs^4^, which still require high-programming voltages (typ. 10 V+)^5^ and their own, dedicated area on the chip, thus forcing the memory element and the circuit using said memory to remain far apart (with all parasitic capacitance and energy dissipation consequences this entails).

The rich landscape of modern electronics design became even more diverse with the steady introduction of memristive devices^6^ into the family of standard electronic components^7,8^. The ability of memristors to act as thresholded electrically tuneable^9^, multi-level^10^, non-volatile resistive loads^11^, combined with their inherently scaling-friendly^12,13^, low power^14^ and back-end integrable^15^ fabrication processes has rendered them a highly promising candidate for use in future electronics applications^9,16–18^. These properties promote memristors as ideal candidates for achieving in silico reconfigurability in a post-Moore context, i.e., without relying on front-end integration density for performance and operating on the principle of separate, dedicated memory and processing elements.

In this work, we lay the foundations of a design approach that amalgamates the analogue non-volatile memory capacity of metal-oxide memristors with the fundamental building blocks of digital design: logic gates. This fusion occurs at a fundamental component level, enmeshing memristors and transistors in order to achieve collocation of memory and computation. Our approach is thus distinct from conventional mixed-signal design; whereby, the analogue/digital parts remain separate entities, interacting purely at the signal level. This true coalescence of paradigms engenders a distinct set of fundamental building blocks: analogue reconfigurable gates featuring embedded memory. We first demonstrate the reconfigurability modes of a memristor-enhanced inverter by delineating how tuning the memory states of individual devices enables the control of the transfer characteristics of the gate. The proposed design paradigm is completed by introducing appropriate readout circuits that make our modified gates interoperable with standard digital gates. We envision this emerging concept becoming a staple in numerous emerging applications and showcase the versatility of the proposed paradigm by experimentally demonstrating two applications: a hardware analogue gate and an analogue domain template matcher.

## Results

### Reconfigurable analogue gate concept, operation and performance

**Fig. 1:**
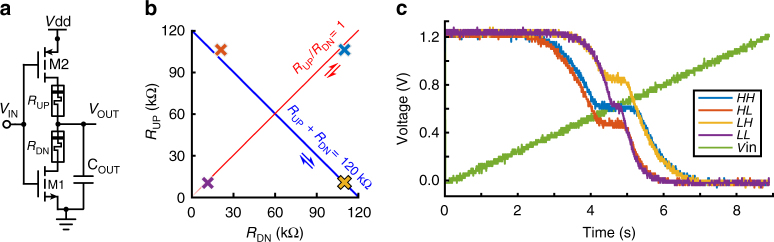
Reconfigurability modalities in an analogue inverter gate. **a** Memristor-enhanced analogue inverter topology. **b** Changing the resistive states of the memristors *R*_UP_, *R*_DN_ in the inverter so as to keep their sum (along blue line) or ratio (along red line) constant offers flexibility in controlling the inverter’s transfer characteristics. The constant sum modality allows independent control of transfer characteristic’s plateau height while the constant ratio modality allows for independent control of the plateau’s width (see Supplementary Fig. 4). Colour-coded crosses correspond to the *R*_UP_, *R*_DN_ configurations used in the results of **c** (see Supplementary Table 2 for details). **c** Four measured examples of analogue inverter transfer characteristics corresponding to the cases where *R*_UP_ and *R*_DN_ are both high (*HH*), high and low (*HL*), low and high (*LH*) and both low (*LL*), respectively. The measured input voltage during the *HH* trial is shown in green as *V*_in_ (similar for all trials). Note independent modulation of plateau width and altitude by the sum and ratio between *R*_UP_, *R*_DN_

Much like multi-valued logic is a generalisation of standard Boolean logic, the proposed analogue gates are inspired as generalisations of standard logic gates. This is realised through the topology shown in Fig. 1a for the analogue inverter (comparison with a standard Boolean inverter is provided in Supplementary Fig. 1). Every current path to/from the output node of the gate is regulated by the presence of a tuneable resistive element, in our case a metal-oxide memristor (see Supplementary Note 1 for the fully general approach). This architecture has the same inputs and outputs as a standard gate but receives analogue inputs and generates an analogue output. Overall, the proposed analogue inverter serves as a potential divider comprising two transistors and two memristors (2T2R). Depending on the precise levels of the input voltages, the output voltage behaviour may be dominated either by the states of the transistors (standard logic gate operation) or by the memristive components and their interrelations (divider operation). The former is obtained when input voltages are clear binary values, while the latter at intermediate levels. This occurs because at each edge of the input voltage range one of the transistors always exhibits a source-drain impedance that is sufficiently high to dominate the entire divider and lead to standard Boolean inverter operation. Simultaneously, at intermediate values of input voltage both transistors are open and the memristive potential divider becomes dominant. This introduces a plateau in the transfer characteristic of the inverter, visible in Fig. 1c, which controls the shape of the mapping between input and output voltages while maintaining the fundamental inverter nature of the circuit (0 maps to 1 and vice versa). Controlling the resistive states of *R*_UP_ and *R*_DN_ allows this soft mapping to be reconfigured. While our design approach is technology-agnostic, the reconfiguration quality is defined by the characteristics of the employed memristive technology. Most importantly, the programming of *R*_UP_ and *R*_DN_ can be achieved by a time-shared scheme via peripheral circuitry that minimises the pixel’s power and area constraints to a minimum; details provided in Supplementary Note 2.

A key feature of the proposed design is its power efficiency that can be illustrated by comparing an analogue vs a digital inverter. For any given input voltage both inverters can be described to some approximation as two component potential dividers, illustrated in Supplementary Fig. 2b. Whenever the input voltage changes from some value *V*_IN,1_ to *V*_IN,2_ the corresponding outputs must change from some *V*_OUT,1_ to *V*_OUT,2_; an operation that requires changing the amount of charge stored on capacitor C_out_ through a capacitor current *i*_cap_ while keeping leakage current *i*_leak_ low (illustrated in Supplementary Fig. 2 with full derivation in Supplementary Note 3). In the case of a standard inverter the only possible input (output) voltages are GND and VDD for logic 0 and 1, respectively, which guarantees that one of the transistors M1 and M2 will always be OFF, i.e., at very high-source-drain impedance. This, in turn, minimises *i*_leak_ allowing the inverter to operate closer to the theoretical lower bound energy ${\mathrm{C}}_{{\mathrm{out}}}{\textstyle{{{\mathrm{VDD}}^2} \over 2}}$ for each state change^19^ (see caveats in Supplementary Note 3). The analogue inverter is governed by the same fundamental dynamics with the exception that M1 and M2 may both be partially ON at the same time. As a result, the inverter becomes capable of performing analogue-in/analogue-out computation at a fraction of the energy consumption of its digital counterpart (e.g., factor of ~4–5 see Supplementary Note 4 for details).

Given any fixed set of resistive states for *R*_UP_ and *R*_DN_, an analogue gate will implement a specific soft input/output mapping, with each memristor constituting a design degree of freedom (dof). Since every memristor augments the impedance seen from the output node to either supply or ground along a unique current path, these design degrees of freedom are linearly independent. Thus, the proposed analogue inverter features two degrees of reconfigurability freedom, whose span is bounded by the range of resistive state values that the corresponding memristor can attain. The design of the CMOS counterpart of the system, and in particular the settings of the key design parameters of transistor aspect ratio ${\textstyle{W \over L}}$ and power supply voltage VDD, may then be tailored so as to optimise gate functionality given the chosen memristor technology’s inherent resistive state ranges (see Supplementary Fig. 3 and Supplementary Table 1). The two-dof reconfigurability space of the inverter is illustrated in Fig. 1b, showing two useful, orthogonal analogue inverter mapping control modalities. In both cases, the values of the two memristors *R*_UP_, *R*_DN_ are altered simultaneously first under the constraints ${\textstyle{{R_{{\mathrm{UP}}}} \over {R_{{\mathrm{DN}}}}}} = c$ (ratio-fixed modality) and then under the constraints *R*_UP_ + *R*_DN_ = *c* (sum-fixed modality), where *c* is a suitable constant in each case. The two control modalities exert orthogonal effects on the plateau in the inverter’s transfer characteristic (Fig. 1c). The ratio-fixed modality controls the breadth of the plateau by altering the balance between the total memristor-transistor (source-drain) impedance while the sum-fixed modality controls the altitude of the plateau by altering the voltage distribution within the memristive potential divider. Provided that the distribution of voltage between memristors is not affected by the overall voltage drop across them, these modalities do not interact with each other (see Supplementary Fig. 4). Fig. 1c presents experimental evidence of an analogue inverter whose memristors have been successively programmed into four configurations: {*R*_UP_,*R*_DN_} ∈ {*HH*,*HL,LH,LL*} where *H*,*L* stand for high and low resistive state, respectively. Results demonstrate operation under an ON/OFF resistive state ratio of ~10, leading to significant changes in the input/output transfer characteristic of the inverter. The *HH-LL* pair illustrates modulation of plateau width independent of altitude whilst the *HL-LH* pair illustrates altitude modulation sans width modulation.

### Interoperability with standard digital electronics

No electronic system can become commercially competitive vis-a-vis standard CMOS technology if it cannot be both read in a simple and efficient manner and modularly chained, which in our case means that the output of an analogue gate has to be a suitable input for the next one. Since, the proposed gates employ analogue voltages as both inputs and outputs this compatibility is ensured. We note however, that with each memristor-based gate acting as a non-linear amplifier, each device maps a relatively restricted range of input voltages to the full power supply range, thus the chaining cannot be continued ad infinitum. Rather it is envisaged that short chains (perhaps 2–5 links) of these analogue gates ending in a digitisation stage, where a 1 or 0 answer will be committed to, will be used. In this manner the ability of digital to perform implicit error-correction by collapsing all answers to a binary space is balanced with analogue’s ability to discern between those two levels. The chaining ability is exemplified in Supplementary Fig. 5, where a NAND gate operates on the basis of input received from an analogue inverter.

**Fig. 2:**
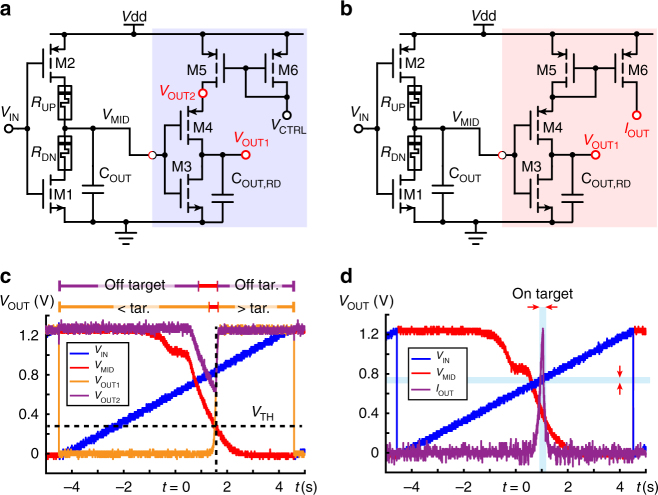
Reading analogue gates. **a** Readout circuit (shaded blue) designed to receive an analogue gate output (*V*_MID_) and then digitise to 1 or 0 based on whether *V*_IN_ is above or below some threshold *V*_TH_ (at *V*_OUT1_) and also indicate whether *V*_IN_ is close or far from *V*_TH_ (at *V*_OUT2_). **b** Alternative readout circuit (shaded red) where *V*_OUT2_ has been replaced by *I*_OUT_, which now indicates proximity of *V*_IN_ with *V*_TH_ by sourcing a large current only if *V*_MID _≅ *V*_TH_. **c** Measured results from the readout circuit in **a**. V_OUT1_ successfully digitises the analogue inverter output *V*_MID_ through most of its input voltage range (*V*_IN_) as shown by the orange bar above the plot (red segment indicates potentially ambiguous digitisation). *V*_OUT2_, on the other hand exhibits a dip only when *V*_MID_ is sufficiently close to *V*_TH_, directly indicating whether *V*_IN_ is on-target (i.e., close to *V*_TH_) or off-target. This is illustrated by the purple bar above the plot. **d** Measured results from the readout circuit in **b**. Only *I*_OUT_ shown for clarity. *I*_OUT_ peaks within a narrow range of input voltages satisfying *V*_MID _≅ *V*_TH_ (on target)

Transferring from analogue logic to Boolean can be easily achieved using a readout circuit consisting of a simple inverter fed through a mirror supply as shown in Fig. 2. The analogue-to-Boolean link rests on the fact that the readout inverter will be characterised by a switch-point voltage, i.e., an input voltage level at which both transistors are simultaneously ON and the inverter output voltage is close to the middle of the supply. Any input voltages above switch-point will be digitised to 0 while values below switch-point will digitise to 1 (Fig. 2, *V*_OUT1_). A small range of input values very close to switch-point, however, will lead to an unclear digitisation that may stochastically result in digital 1 or 0 values. Notably, changing the resistive states of the memristors in any analogue gate can alter the input variable space regions which lie above/below the readout inverter switch-point and thus indirectly tune the overall mapping from analogue gate inputs to digital output. The switch-point of the readout inverter is determined by the aspect ratio (*W/L*) of its constituent transistors and is a design parameter; though there is no reason memristors cannot be used to render the readout inverter reconfigurable, too. On the other hand, converting Boolean-to-analogue requires no conversion as any Boolean input level is automatically a valid analogue input.

The mirror supply shown in Fig. 2a is not strictly necessary for the digitisation strategy described previously, but offers an interesting alternative approach. The principle of operation relies on the observation that the readout inverter conducts most current at the switch-point voltage, when both its transistors are maximally ON simultaneously and digitisation at node *V*_OUT1_ is unclear. The mirror supply exploits this by attempting to force a reference current into the inverter. If the reference current is chosen appropriately, the voltage on node *V*_OUT2_ from Fig. 2a is driven towards a digital 0 only when the inverter is sufficiently close to its maximally conducting state (both transistors simultaneously open). This circuit therefore offers a way of performing digitisation by mapping a very-specific analogue gate output level to a digital 1, i.e., allowing that particular value to act as a target and the analogue gate to output an off-target/on-target response (see 2c, purple bar). Modifying the magnitude of the reference current will tune the analogue gate output level range for which digitisation using this strategy will return a digital 1. A slight modification of the mirror supply leads to the circuit depicted in Fig. 2b, which mirrors the readout inverter current directly to a circuit terminal. Providing the digitisation output in the form of a current allows for easy summation of digitisation results from many analogue gates. The resulting, summed current can then easily be provided as input to an integrate and fire neuron as used in neuromorphic engineering^20^. Notably, both variants in Fig. 2a, b indicate that the on-target range of the input voltage is of the order of 100 mV as evidenced by *V*_OUT1_ voltages that are no longer a clear digital 1 or 0 (Fig. 2a, c) or *I*_OUT_ currents significantly above baseline (Fig. 2b, d). The on-target range will depend on the resistive state values of the memristors as is immediately evident by observing that the traces in Fig. 1c will intercept the threshold shown in Fig. 2c at different points along the *x*-axis (and consequently different input voltage levels).

### Case studies: an analogue gate and a template-matching pixel

**Fig. 3:**
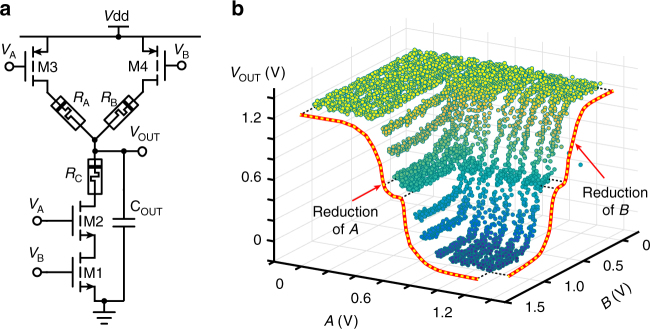
Analogue NAND architecture and basic behaviour. **a** Analogue NAND topology. **b** Measured analogue NAND transfer characteristics. The inverter reductions of A and B (see text) are shown as red/yellow lines. The overall transfer characteristic arises from an essentially multiplicative relation between the reductions of A and B

The introduced design paradigm forms a generalised framework that can be used to develop a broad range of applications. Here we showcase two cases, namely: analogue gates and template matching. Soft logic relies on analogue-in/analogue-out gates in order to perform computation on a continuum between the extreme values of 0 and 1. This implements a soft input/output mapping that can be used for function approximation. The example of an analogue NAND gate is shown in Fig. 3. An analogue NAND can be implemented via a three-way divider consisting of four transistors and three memristors (4T3R). Figure 3b shows the transfer characteristics from the two inputs to the output, which now define a surface. Notably, when input A is fully ON (digital 1), then the analogue NAND reduces to an analogue inverter from B to the output with a mapping determined solely by memristors M_B_ and M_C_. We shall term this the analogue inverter reduction of B. The same holds when input B is a digital 1. In the case where either of the inputs is fully OFF (digital 0), the output of the analogue NAND will always be a digital 1. Changing the resistive states of the memristors controls the shape of the analogue function surface while retaining its inherent NAND nature. The measured results shown on Fig. 3b denote an essentially multiplicative interaction between the analogue inverter reductions of A and B, suggesting that the analogue function surface may be controlled in a reasonably orthogonal way by varying M_A_ and M_B_ at the cost of restricting the possible shapes it may assume. Similar conclusions may be drawn for other types of gates (see Supplementary Fig. 6), although analogue NANDs are already functionally complete (in the sense that using multiple analogue NANDs, mappings corresponding to any other analogue gate can be constructed).

**Fig. 4:**
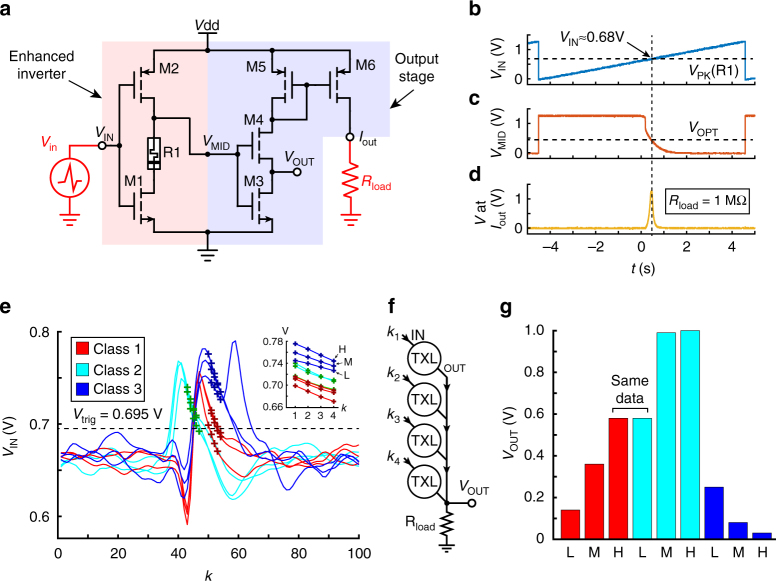
Analogue domain template matching enabled by memristive technologies. **a** Schematic of texel circuit illustrating consisting of an analogue inverter and the readout stage (red and blue shading correspondingly). **b**, **c, d** Texel transfer characteristics from input voltage (**b**), through mid-point voltage *V*_MID_ (**c**) and voltage at the output node (**d**) marking *V*_IN_ input voltage level (*V*_PK_) and *V*_MID_ voltage level (*V*_OPT_) at which the output stage sources its maximum current. **e** Selected spike waveforms used as input to the test texel array. Crosses indicate the sample points used to feed the array. *k*: sample number. *V*_trig_: texel array sampling trigger level (Methods section). Inset: close-up of the chosen sample points. L, M, H and arrows: Low, medium and high-voltage instances of spikes in class 3. **f** Schematic of 4-texel array used to carry out experiments. **g** Measured output voltage when spike samples from **e** are applied to the texel array in **f**. L, M, H versions of spikes in each class shown. Higher *V*_OUT_ voltage means greater degree of matching between input data and stored template. The texel array was programmed to respond best to class 2 spikes. Colours as in **e**. Class 1-H and class 2-L results refer to the same experiment (Methods section)

Template matching is a technique whereby a small part of a signal (audio/electrical waveform snippet or even image segment) is compared against a stored template. Specifically in the emerging field of bioelectronics, template matching facilitates neural spike sorting for electrophysiological studies^21^, whereby electrical waveforms recorded from neural cell assemblies are template-matched in short snippets of typically 10–20 samples^22^. Its strength stems from the fact that whenever a match is found the system registers the occurrence of a spike and the matching template ID, thus simultaneously providing spike timing and identification information. A simplified version of our proposed analogue inverter with the readout circuit from Fig. 2b is used herein, shown in Fig. 4a, for demonstrating spike detection/sorting. We refer to this circuit as a texel (template-matching pixel) and its operation can be understood as searching for an input voltage value *V*_IN_ that matches stored value determined by the resistive state of memristor R1. This process is shown in Fig. 4b,  d by illustrating measured transfer characteristics of a discrete texel. This implementation, being modular, power efficient and truly scalable, allows for aggregation into arrays; a proof of concept 4-texel array is further shown in Fig. 4f. The array is fed with appropriately selected samples (Methods section) from nine neural spike waveforms from the same database^23^ (Supplementary Fig. 7) and summing the current outputs of each texel down a common load resistor, as shown in Fig. 4e, f. Three spike instances were chosen from each available class of spikes: a low (L), a medium (M) and a high (H) instance corresponding to spikes exhibiting lower than, similar to or higher than class-average voltage levels (Methods section and Supplementary Fig. 7). The voltage level at the system’s *V*_OUT_ terminal is linked to the degree of matching between the input vector k and the stored template and was directly used as a matching degree metric. Due to the similarity between the H instance of class 1 and the L instance of class 2 and the limited resolution of our instrumentation, the experiment for these two instances was ran only once with a common input vector k (see Supplementary Table 3). Results in Fig. 4g show a texel array setup to discriminate for class 2 spikes. Even using only four samples from each waveform (marked in Fig. 4e) strong discrimination between templates is clearly achieved. The memristor resistive states were confirmed to remain stable before and after the experiment (Supplementary Table 4).

## Discussion

Our design approach provides a simple, powerful and generic tool for truly fusing the analogue and digital design worlds. We note that this concept is optimised first for low energy dissipation and then for speed, reflecting the pertinent needs of modern ubiquitous computing hardware. We thus envision the proposed technology to complement and coexist with standard CMOS implementations. The transistors and memristors in Fig. 1a can in fact be any elements that modulate a resistance (e.g., floating gate MOS), however, our proposal of embedding memristors as analogue resistance tuning elements yields a power efficient implementation. In every case, the basic concept remains the same: signal-controlled tuneable resistance elements intended to operate as ON/OFF switches are combined with, initialise and operate continuously tuneable resistance elements. This combination results in systems where digital and analogue behaviours coexist, with the ON/OFF and continuously tuneable components tasked to sustain each type of operation respectively. Importantly, we consider the reconfigurability aspect of our approach to be of fundamental significance as it can endow embedded hardware with the capabilities of (1) trimming to compensate for technology (both CMOS and memristors) imperfections (variation and mismatch), (2) tuning their functionality for efficiently addressing applications involving drifting specifications (e.g., texel—searching for a variety of spike templates) and (3) repurposing the core operation of a system.

At a technical level there are some crucial points to be noted: First, though it may seem counter-intuitive to use canonical circuits such as the one in Fig. 1 that include a current leakage term it turns out that because of the sheer simplicity of the circuit some tasks may be carried out more efficiently than even using traditional digital approaches, as shown in Supplementary Note 4. This holds insofar as each of these circuits is operated only for as long as it is computing something useful and coming to a stable answer (i.e., for the correct number of time constants as remarked in Supplementary Note 3). For the rest of the time the circuit may be either power-gated off, or simply parked in a digital state where its inputs are forced to a binary value and the system reverts to standard Boolean operation, resulting in CMOS-comparable leakage.

Second, the issue of variance often plagues analogue electronics, leading to the use of transistor sizes much larger than technically necessary given the rules of how mismatch scales^24^. Introducing trimming elements in our circuits we have a degree of freedom previously not obtainable in pure CMOS alone. Especially, if we consider the case of the  program rarely and read often operating regime, using a trial and error-based programming circuit, as described in Supplementary Note 2, the possibility of automatically compensating for mismatch and variation through tuning of resistive states becomes viable. This is extremely important as it removes one of the biggest barriers analogue electronics face when downscaling. In this work, we have kept transistor sizings conservatively large in order to ensure that even without resistive state tweaking (all devices assumed programmed at nominal) the variance shown by Monte Carlo simulations (shown in Supplementary Fig. 8) remains under check. The overarching aim of this work is to push the limits of scaling by introducing performance-check-based programming. The exceptionally high resolution of memristor resistive states^10^ works in our favour by supporting the idea of very fine resolution, ultra-compact, few-component memristive trimming (no need for digital registers and switching banks). Finally, we note that proving dominance over purely digital approaches is by no means trivial. The fact however that our proposed circuit architectures consist of few relatively large transistors, as opposed to many fully downsized ones, implies that there may still be room at the bottom for additional improvements.

At the application level we note that our template matching example might appear niche to begin with, however, pattern matching in general is one of the most fundamental operations of signal processing. In the case of spike sorting the raw signal itself is such that a template matcher can identify a spike class directly from a simple set of texel inputs. Nevertheless, the input signal need not be a waveform and need not be raw (unprocessed by some other system before entering the template matcher). A simple example could be an output vector from a neural network layer (e.g., a few levels deeper than the input layer).

In conclusion, this work lays the conceptual foundations and provides experimental proof of a design approach for electronics formed by marrying standard, digital circuitry with analogue electronics and rapidly emerging memristive devices to offer analogue computation at close to the power/area price of digital with the added benefit of reconfigurability. This is achieved by collocating and enmeshing memory and computation whilst maintaining full interfacing compatibility both with standard digital logic and internally (ability to chain analogue gates as is done with digital gates). Finally, the versatility of this paradigm is illustrated by two independent applications in analogue logic and template matching for bio-signals that given the power constraints of alternative technologies would have been impossible to realise.

## Methods

### Memristive device fabrication and specification

Memristive device fabrication and specification: All the memristors used in the experimental set-ups are in 3 × 3 mm^2^ chips that are wire-bonded to PLCC68 packages. Each memristor is a 20 × 20 µm^2^ cross-point of top and bottom electrodes (TE & BE). The BEs were first fabricated on 6 inch Silicon wafer that was thermally oxidised to grow 200 nm thick SiO_2_, which serves as insulating base layer. Using conventional optical lithography the BEs were patterned with negative-tone resist AZ-2070 followed by a low power and short reactive ion etching (descum) to remove any residual resist on the patterned areas. Then, 5 nm Titanium (Ti) and 10 nm Platinum (Pt) film were deposited with electron beam evaporation at low rate (0.5 Ås^−1^), with Ti serving for adhesion purposes. Leybold_Lab700eb tool that has high-crucible-wafer distance (greater than 1 m) was used for evaporation to guarantee parallel deposition. The combination of parallel evaporation and negative-tone resist which has undercuts after development ensures a good lift-off process, resulting in well-defined electrodes without wings (fences) that would affect the subsequent thin layer, thus becomes harmful for the final device. Next, similar photolithography was carried out using the active-layer mask that allows depositing the metal-oxide active bi-layer everywhere except on the BE pads, followed by 1 min descum. Lambda controlled plasma assisted reactive magnetron sputtering (Leybold Helios Pro XL) was used to deposit the active layers; 25 nm TiO_2_ followed by 4 nm Al_2_O_3_, at room temperature. TiO_2_ was sputtered from Ti metal target with 8 sccm O_2_, 35 sccm Ar flows and 2 kW at the cathode, and 15 sccm O_2_ flow and 2 kW at an additional plasma source. Al_2_O_3_ was sputtered from Al metal target with 15 sccm Ar flow and 100 W at the cathode, and 25 sccm O_2_ flow and 1.5 kW at the additional plasma source. Before any sputtering, the additional plasma source was used for an extra clean of the substrate with 8 sccm O_2_, 10 sccm Ar and 2 kW. Optiwet-ST30 tool was then used to perform lift-off process with the following parameters; 3 mbar pressure and 60 °C hot NMP for 1 h. This tool ensures 100% lift-off yield even after sputtering which deposits material everywhere making the lift-off difficult, and even at large features (pad areas) that tends to stick to the surface after dissolving the resist. One minute descum is needed to clean the surface before TE lithography. The 10 nm Pt TEs were patterned and defined in similar fashion to the BEs. No sonication was used in this process. Finally, 3 × 3 mm^2^ chips were diced for wire-bonding.

### Experimental set-ups and procedures

All experiments illustrated in Figs 1–4 were carried out on circuits prototyped on breadboard or strip-board. External power supplies and signal generators were used to supply both signal inputs and power whilst results were gathered exclusively by oscilloscope. For these experiments packaged devices were used, connected to the set-ups via breakout boards. We used custom made 64-pin breakout boards connecting the devices to the rest of the system using jumper wires. This set-up is significant because it demonstrates functionality after wafer dicing and wire-bonding. All experiments were ran under a power supply between 1.2 and 1.3 V (single-decimal figure precision power supply). In each case, the memristive devices used were placed in the required resistive states using an ArC ONE instrumentation board (ArC instruments, UK). All devices used for all experiments were located on the same die, i.e., only one memristive device package containing a total of 32 memristors was sufficient to carry out all the work presented here.

Additional notes on analogue NAND experiment: Results for the analogue NAND experiment were taken strip-by-strip by setting input *A* to a succession of fixed values and sweeping input *B* for each of those values by use of ramp (saw-tooth) signals. Input *A* was stepped with 100 mV resolution throughout the entire power supply range except between 0.5 and 0.8 V where it was stepped with 50 mV resolution for enhanced visibility.

Additional notes on spike sorting application experiment: A 4-point texel array with common load resistance of 300 kΩ was implemented. The signals fed into this system arrived from two, dual-channel benchtop power supplies with two significant decimal digits resolution. The benefit of using synthetic neural recording input data is that it contains ground truth information on spike identification and timing. On that basis an automatic sample selection script was ran on each spike instance available in the dataset in order to choose which data-samples from each instance are to be fed into the texel array for matching against a stored template. The script operated as follows: the data-points in each spike instance were read sequentially and once a trigger threshold *V*_trig_ was exceeded for the first time the script skipped six samples and then choose the subsequent four as candidate inputs for the texel array set-up. This methodology was chosen because it rendered the three classes of spikes visibly distinguishable despite the use of only four template points. The overall trigger and sample approach is similar to the work by Restituto-Delgado and colleagues^25^. In a more mature system implementation a larger texel array containing more than four samples would be used. Next, the extracted candidate four-texel sample sets were separated by single unit-template class. From each class, three texel sets were chosen for further processing: one featuring typical (M), one featuring lower than usual (L) and one featuring higher than usual (H) voltage values (selection shown in Supplementary Fig. 7). Waveforms where the presence of more than one spike within each instance had corrupted the output of the sample selection script were automatically excluded from the selection. The voltage range of all nine selected sample sets (L, M, H instances for each of the three classes) was then adjusted by application of a common pair of gain and offset settings (Gain: 0.1; Offset: 0.66 V). The adjusted texel data-point voltages were then suitable for working with the input voltage values the texel circuits were built to discriminate between. These adjusted values are shown in the inset of Fig. 4e and were used as the input to the texel array after being rounded to 10 mV precision (two significant decimal digits). This procedure caused the rounded texel voltages of the H instance of class 1 and the L instance of class 2 to completely overlap, hence that experiment was conducted only once for both cases.

### Data availability

All data supporting this study are openly available from the University of Southampton repository at https://doi.org/10.5258/SOTON/D0525.

## Electronic supplementary material

Supplementary Information
