# Supplementary material for: Seamlessly fused digital-analogue reconfigurable computing using memristors
Source: Nat Commun. 2018 Jun 4;9:2170. doi: 10.1038/s41467-018-04624-8 (PMC5986819; doi:10.1038/s41467-018-04624-8)
Supplement: Supplementary file 1 — Supplementary Information [file 41467_2018_4624_MOESM1_ESM.pdf]

Seamlessly Fused Digital-Analogue Reconfigurable Computing using Memristors

Serb et al.

Supplementary material:

Supplementary figures:

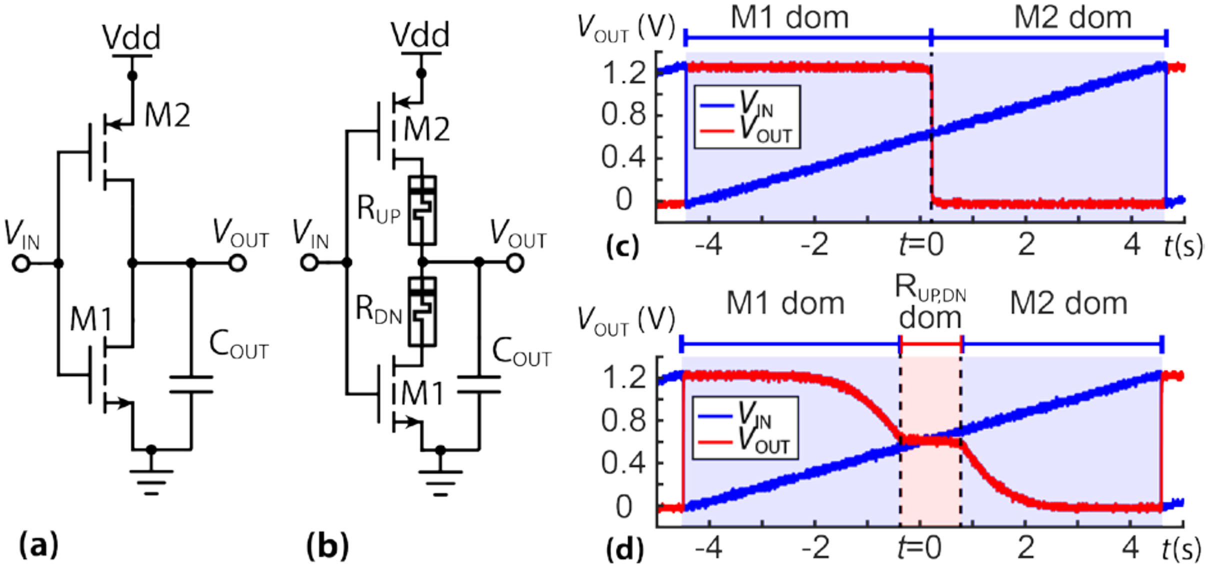

Supplementary figure 1: Analogue inverter gate architecture and basic behaviour. (a) Standard Boolean and (b) memristor-enhanced analogue inverter topologies. (c) Measured Boolean and (d) analogue inverter transfer characteristics. Devices R_{UP} and R_{DN} in the analogue inverter are memristors. The skew in the input/output transfer characteristic introduced by the memristors is evident. In both (c) and (d) blue/red bars above the figures indicate which component's resistance dominates the total impedance of the potential divider formed between Vdd and GND. The red-shaded, plateau region in the transfer characteristic of the analogue inverter shows the range where the memristors provide most of the impedance as transistors M1 and M2 are simultaneously open.

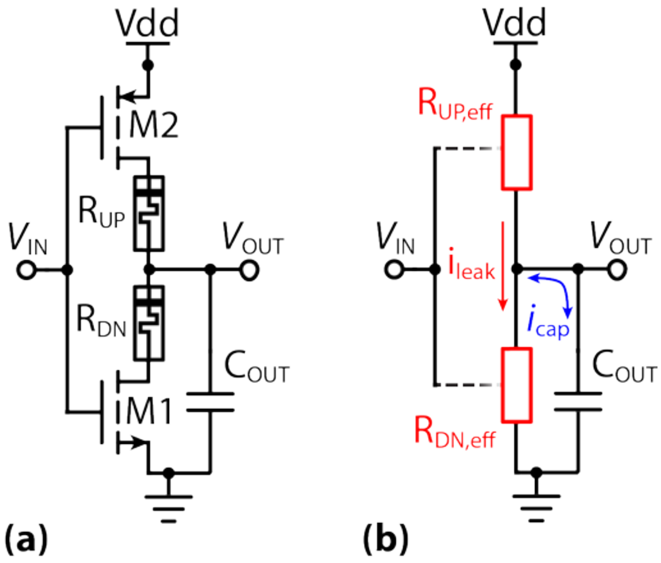

Supplementary figure 2: Simplifying assumption for power estimation analysis. (a) Analogue inverter topology. (b) Simplified version of (a). The combinations of R_{UP}–M2 and R_{DN}–M1 have been modelled as effective resistances $R_{UP,eff}$ and $R_{DN,eff}$ for fixed V_{IN}. Moreover, the key current components i_{cap} and i_{leak} are illustrated.

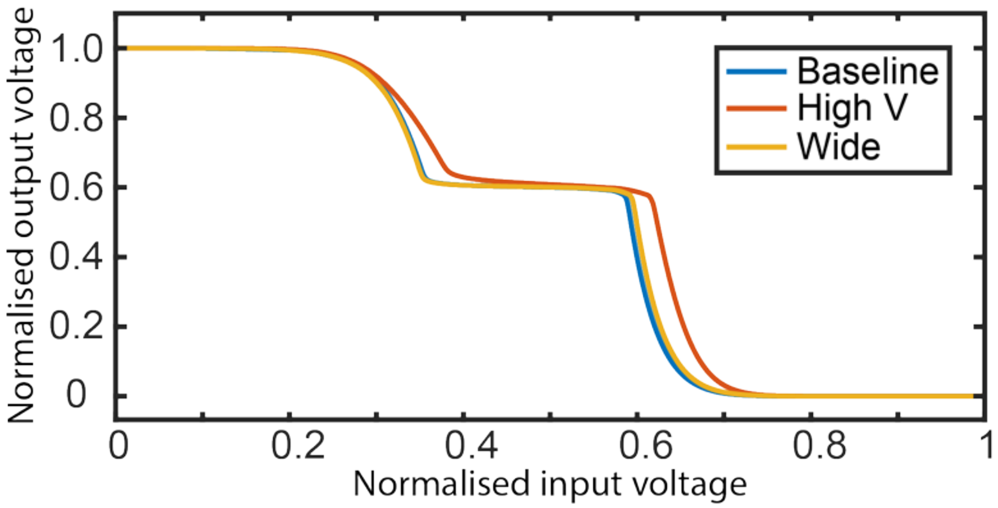

Supplementary figure 3: Trading off transistor sizings and power supply voltage against memristor resistive states. Three analogue inverter (Figure 1b) input/output transfer characteristics are shown, where two cases, 'High V' and 'Wide' are compared against a baseline case. In the 'High V' case the memristors operate at one order of magnitude lower resistive state vs. baseline and the power supply has been increased from 1.65V to 2.2V to compensate. In the 'Wide' case, the memristors also operate at one order of magnitude lower resistive state vs. baseline, but the inverter transistors have been increased tenfold to compensate. Interestingly, increasing the power supply leads to a slight shift in the location of the plateau, possibly because of the asymmetry between pMOS and nMOS characteristics. Notably, however, the power supply voltage can be chosen such that the plateau width remains the same. Detailed set-up parameters for each case can be found in Supplementary Table 1, including transistor specifications.

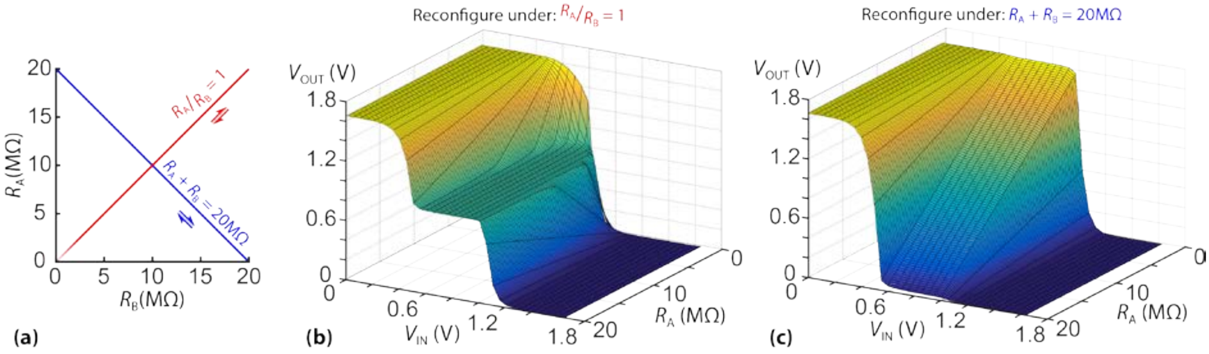

Supplementary figure 4: Reconfigurability of an analogue inverter akin to the one shown in Figure 1a. (a) Reconfigurability space demonstrated in (b) and (c), covering constant sum (=20MΩ) and constant ratio (=1) cases. (b) Constant ratio case. The plateau widens as R_A (and consequently also R_B) increases. (c) Constant sum case. The altitude of the plateau decreases as R_A increases (and consequently R_B decreases). Transistor specifications as in baseline case of Supplementary Table 1.

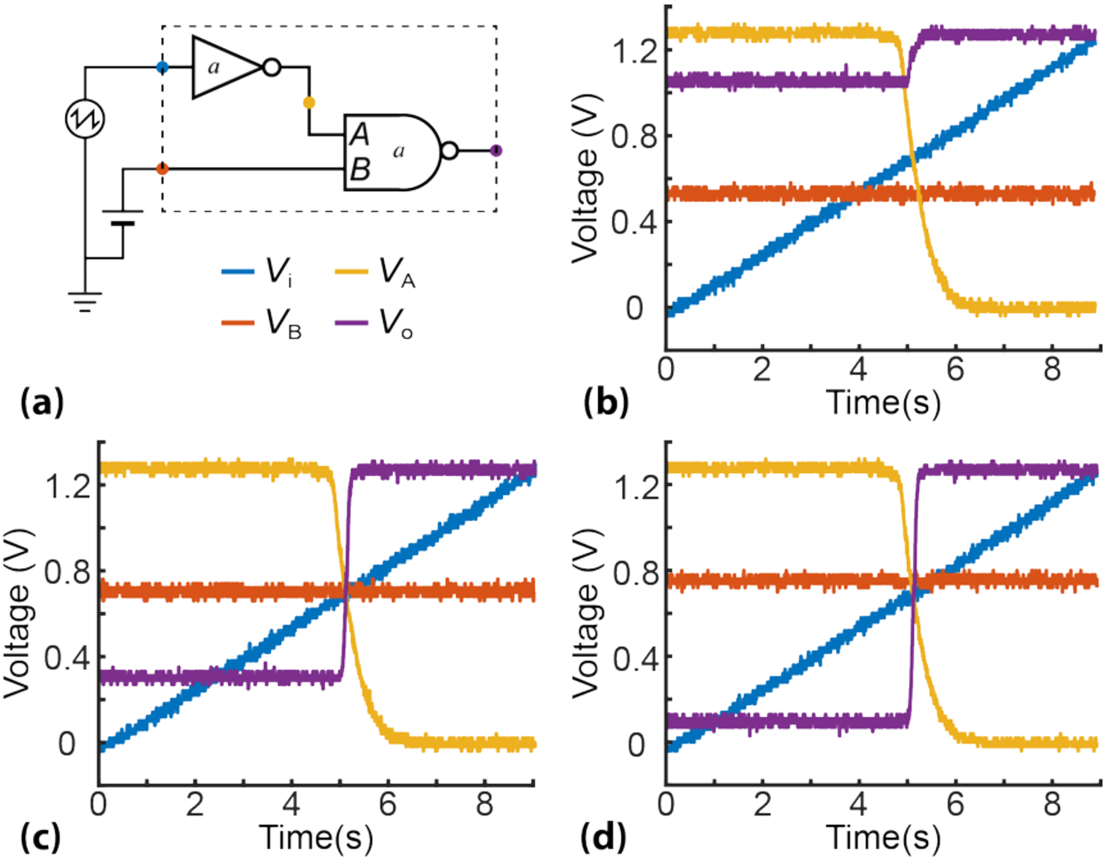

Supplementary figure 5: Example of analogue gates working together. (a) Topology tested: an analogue NAND gate with one input fed directly from a fixed voltage source and the other output fed from a saw-tooth source via an analogue inverter. (b-d) Three measured examples of the system input/output transfer characteristics, each taken for a different value of V_B. We notice that in (b), where V_B is lowest the system always evaluates the function $\overline{A \cdot B} = \overline{\imath \cdot B}$ as fairly true (V_O close to VDD). As the value of V_B is increased, however, the system evaluates the same expression as less and less true. This occurs until V_A drops below approx. 0.7V, in which case V_O reaches VDD across all panels (b-d).

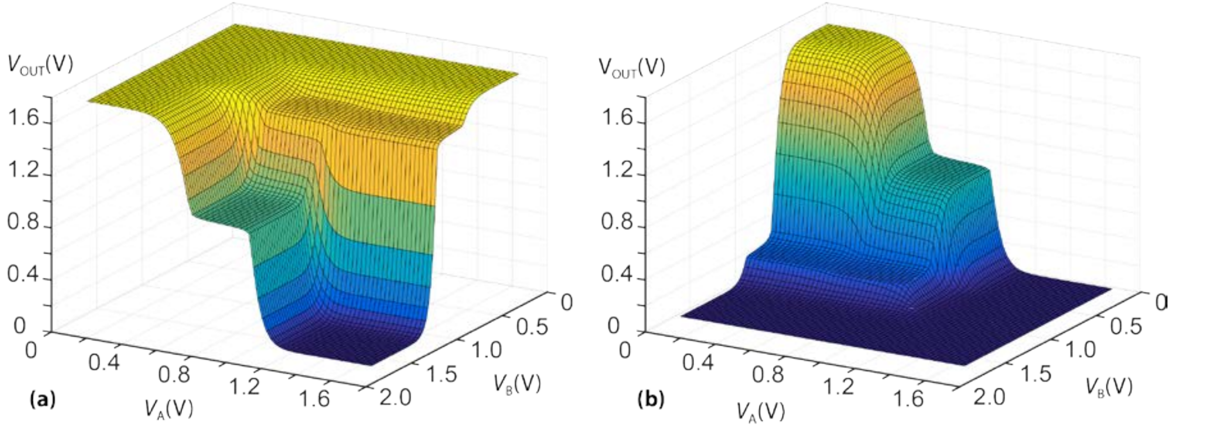

Supplementary figure 6: Demonstration of analogue NAND and NOR gates. (a) NAND gate input/output transfer characteristics. Schematic as shown in Figure 3a. R_A = 3.5kΩ, R_B = 0.5kΩ, R_C = 4.0kΩ. (b) NOR gate input/output transfer characteristic. This gate is the exact dual of the NAND gate used for (a) (Exchange the power supplies to turn NAND into NOR and vice versa) and employs the same values of resistive states. pMOS/nMOS transistor specifications as in baseline case of Supplementary Table 1.

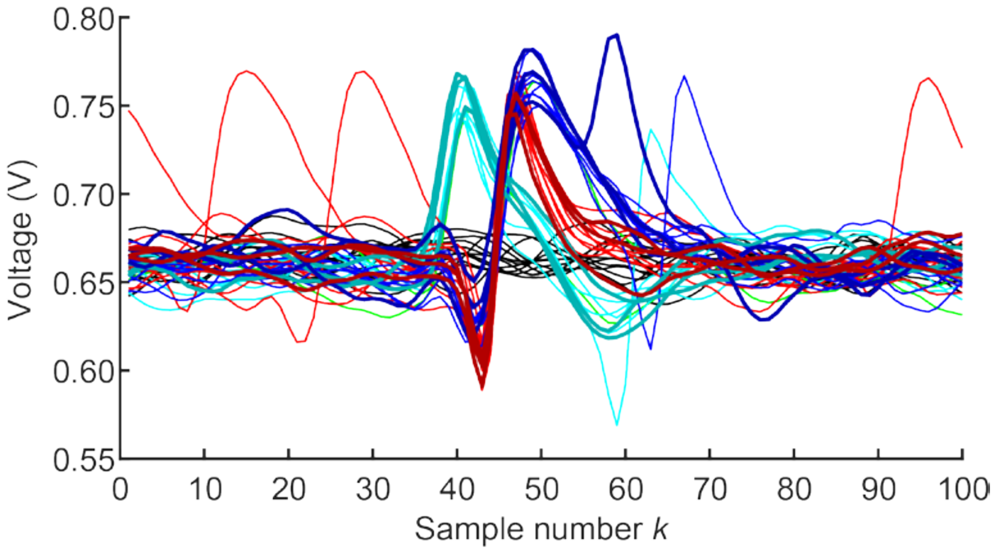

Supplementary figure 7: Dataset used for carrying out texel array experiment in Figure 4. Shown are all neural spike waveforms included in the dataset with colours indicating their class (same colour scheme as Figure 4). Spike waveforms chosen as inputs for the experiment in Figure 4 are shown as thicker, darker traces.

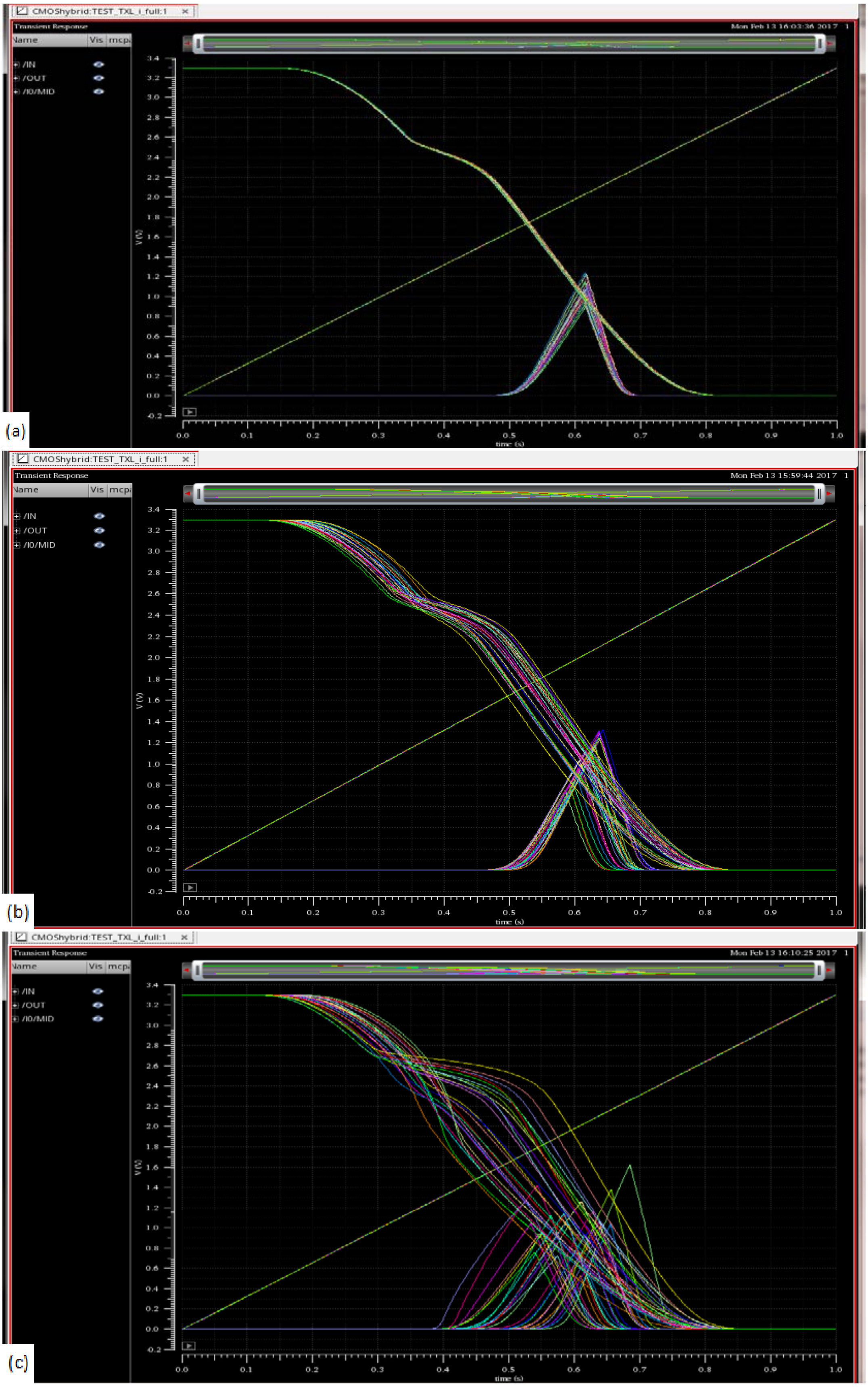

Supplementary figure 8: Monte Carlo simulations on an analogue inverter. Shown are: the input voltage over time (straight line), the inverter transfer characteristic, i.e. the output voltage over time (monotonically decreasing curve) and the output current converted into a voltage via a sense resistor (peaking waveform). 100 Monte Carlo runs in each case.

(a) Mismatch only variation – fix using good layout techniques and trimming memristors. (b) Process variation only – potentially fixable using global controls (e.g. PS modulation – outside the scope of this work) and/or memristor trimming. In both (a) and (b) the memristors are replaced by resistors that operate at nominal resistance simulating the effect of blindly, but accurately programming the devices to their nominal (not necessarily performance optimum) level. This is demonstrably achievable by use of appropriate pulsing schemes[1,2]. (c) PV and mismatch if memristors are replaced by polysilicon resistors. System is not viable without memristors.

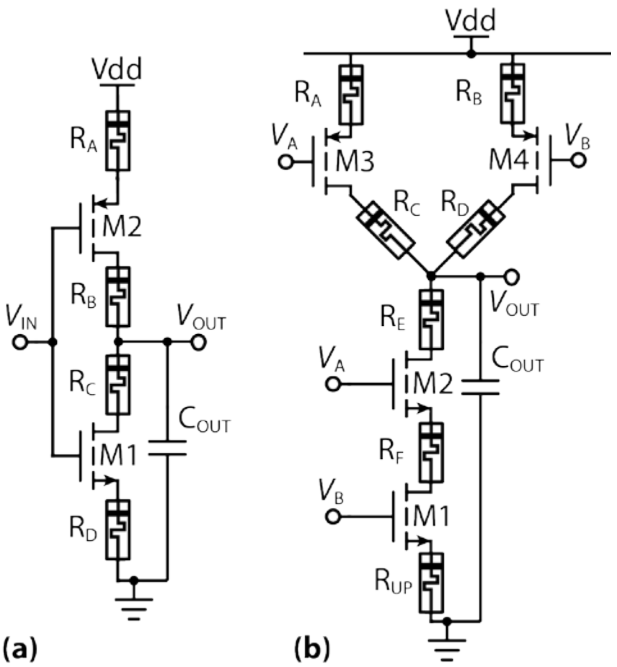

Supplementary figure 9: Fully generalised memristor-based analogue gates. (a) Fully general analogue inverter. (b) Fully generalised NAND.

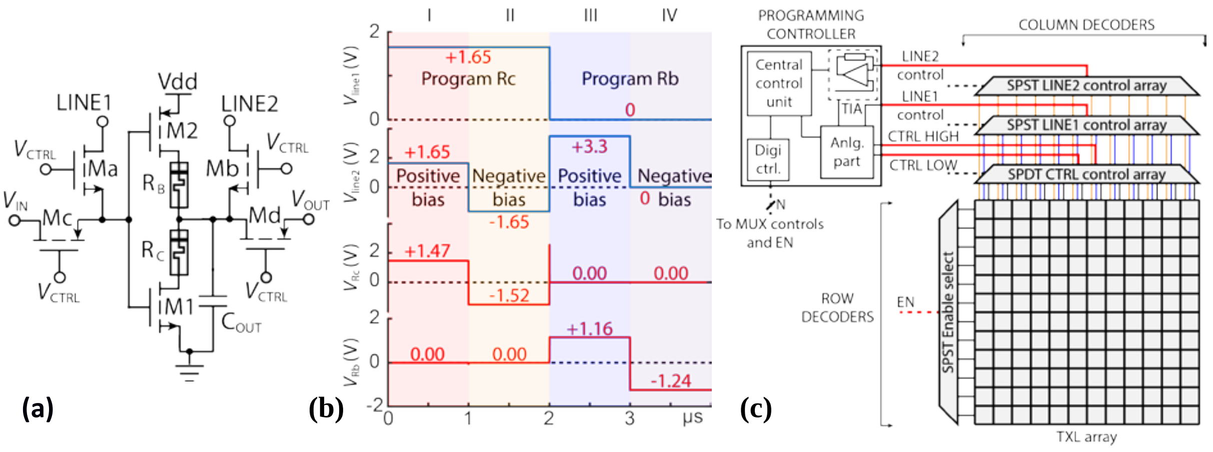

Supplementary figure 10: Programming infrastructure. (a) Gate-level infrastructure consists of four NMOS transistors controlled by V_{CTRL}. When it is active, then the system is in programming mode, the output of the gate is isolated and so is the input and the voltages at both input and output nodes of the inverter are determined by LINE1 and LINE2. (b) Simulations showing the ability of the programming circuit to impose greater than 1V voltages in both polarities on both devices. The four phases (I-IV) correspond to the possibilities of programming both memristors R_B and R_C in both polarities as indicated in in the top half of the figure. Bottom half of figure shows the voltages applied at both memristive devices throughout the four phases, clearly indicating that the devices are being programmed independently. R_A = R_B = 100kΩ, V_{CTRL} = +4.5V. V_{IN} = V_{OUT} = VDD. No additional C_{OUT} was introduced. All transistors are minimum size except pMOS device that feature W/L = 0.7/0.35 microns. (c) System-level architecture showing row and column decoders and programming control unit.

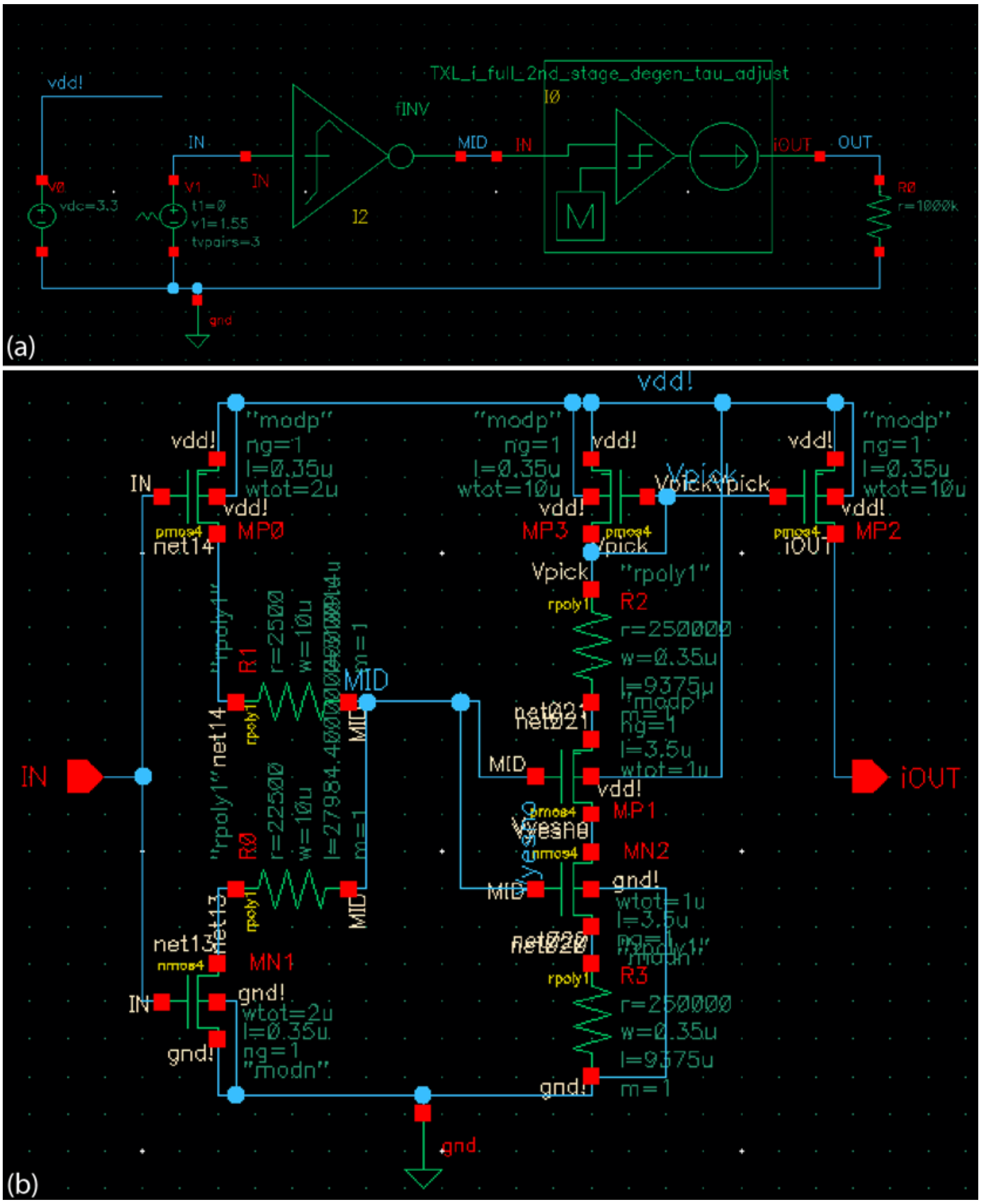

Supplementary figure 11: Schematics of texel power dissipation test bench (a) and texel circuitry (b) used to carry out power dissipation simulations. The driving inverter in (a) is similar to the inverter in (b), i.e. devices MP0, MN1, R0 and R1. In (b) memristors are represented by resistive elements. R0 and R1 are the memristors tuning the transfer characteristics of the texel whilst R2 and R3 are optionally implemented for tuning the sensitivity of the output current to input voltage and to act as current limiters. These memristors need not switch after fabrication and act as simple resistive loads.

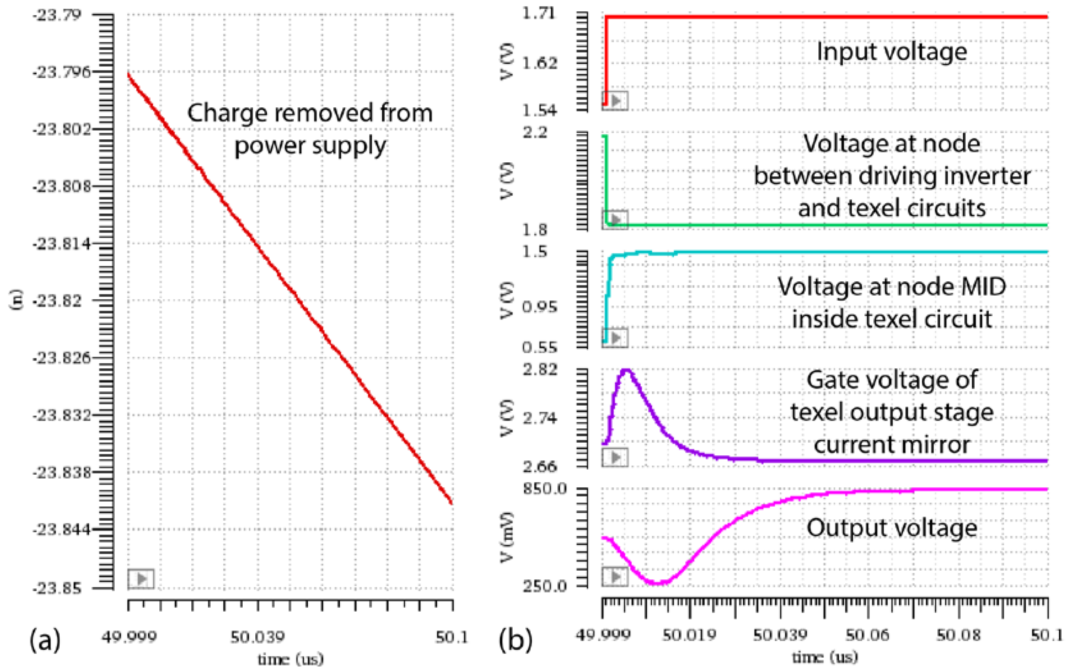

Supplementary figure 12: Charge dissipation of the test bench circuit in Supplementary Figure 11a for input signal transition from 1.55V to 1.70V. AMS 0.35 micron technology with power supply set to 3.3V. Approximately 46fC escape the power supply throughout the process, corresponding to toggling 37 minimum drive strength inverters as shown in Supplementary Figure 13. The design under study has not been optimised for power. (a) Charge dissipation through test circuit over time. (b) Selected voltage signal time evolutions from system input (red trace) to system output (green trace).

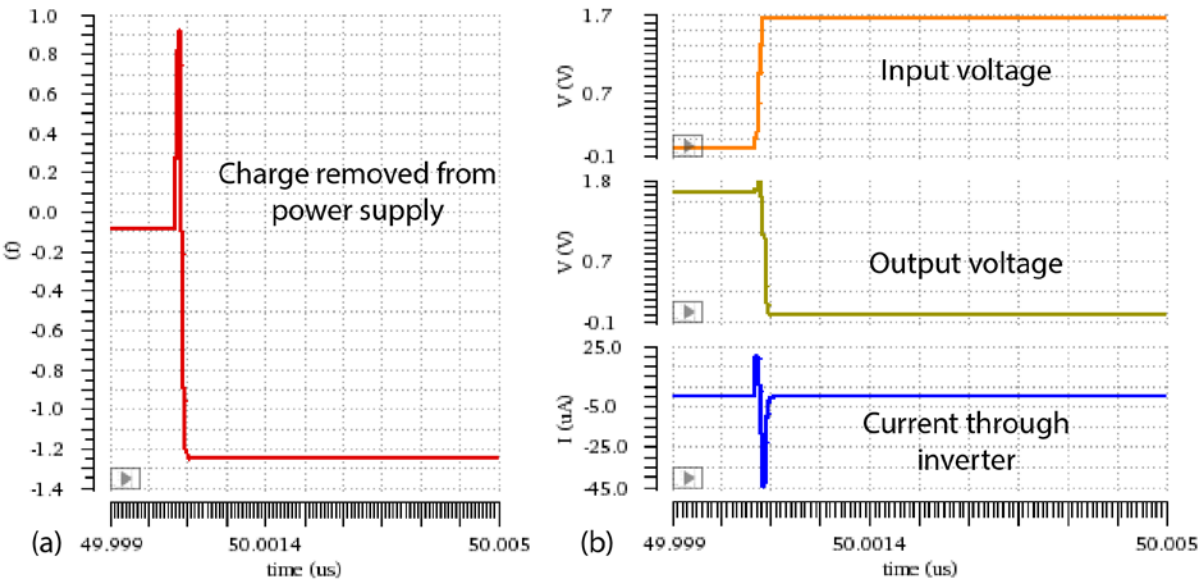

Supplementary figure 13: Charge dissipation of a minimum strength inverter in AMS 0.35 micron technology for a single input signal toggle. Approximately 1.25fC escape the power supply through the process. VDD = 3.3V. (a) Charge removed from power supply vs. time. (b) Input voltage signal. (c) Output voltage. (d) Current through the inverter.

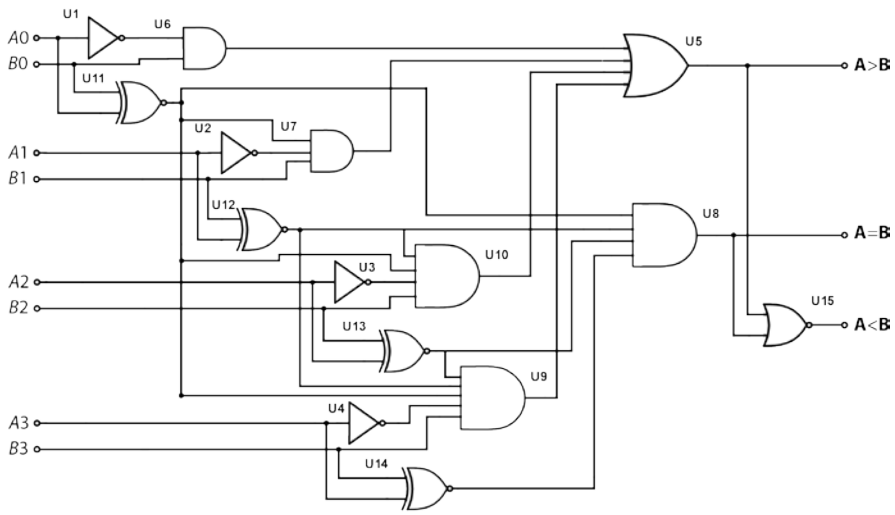

Supplementary figure 14: A 4-bit digital comparator. Two of these can be used in order to carry out a range comparison. The input value may be presented e.g. as digital vector A = {A_0, A_1, A_2, A_3} whilst the reference value can be presented in digital vector B.

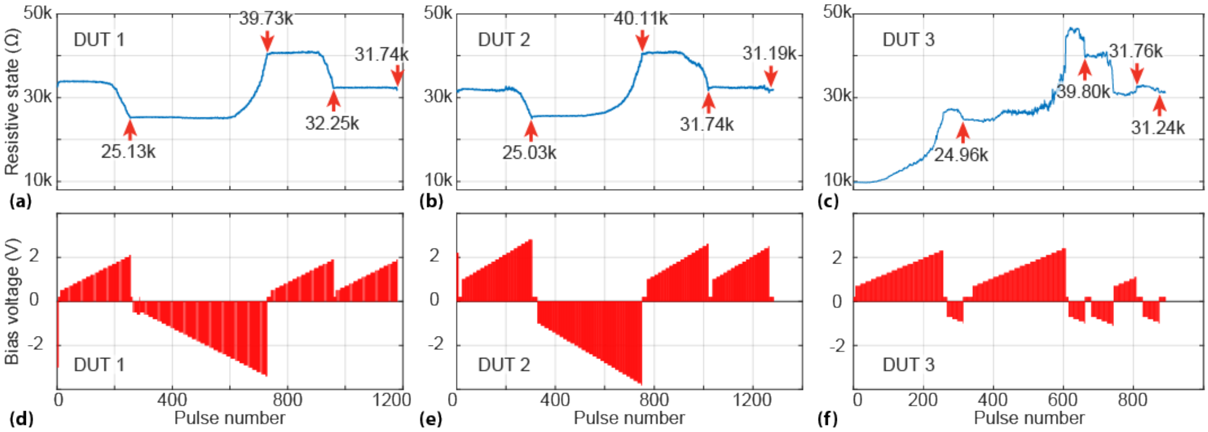

Supplementary figure 15: Measured examples of controllability of memristive device resistive states. Three devices are shown as they are cycled by an automatic 'set to user-defined resistive state' algorithm through a schedule with target values [25kΩ - 40kΩ - 32kΩ - 31kΩ]. (a-c) Resistive state evolution for devices under test (DUTs) 1, 2 and 3 respectively. (d-e) Corresponding voltage stimulation. Read pulses at 0.2V are observed between the incremental step pulse train ramps.

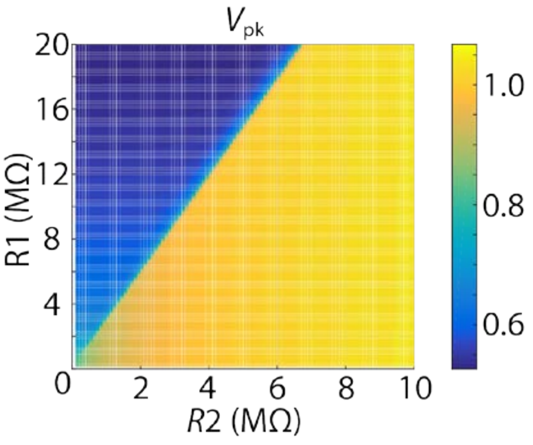

Supplementary figure 16: Prospective range of operation of a texel circuit as shown in Figure 2b simulated to operate under a power supply of 1.65V. *Vpk* represents the input voltage that maximises output current. The results illustrate that our introduced texel can be employed with a variety of memristive technologies that utilise broader ranges of resistive states from what has been experimentally verified in this work.

Supplementary tables:

	Baseline	Wide	High V
**W2 (µm)**	12	120	12
**L2 (µm)**	3.5	3.5	3.5
**RUP (kΩ)**	4000	400	400
**RDN (kΩ)**	6000	600	600
**W1 (µm)**	1	10	1
**L1 (µm)**	3.5	3.5	3.5
**VDD (V)**	1.65	1.65	2.2

Supplementary table 1Parameters used for simulations demonstrating the trade-off between power supply voltage transistor sizing and memristive device resistive states. See Supplementary Figure 3.

	R_{UP} (kΩ)	R_{DN} (kΩ)
HH	106	110
HL	106	20.9
LH	10.5	110
LL	10.5	11.5

Supplementary table 2: Resistive states of memristors, as measured under standard read-out voltage of 0.2V. Used for the experiment in Figure 1.

	**SPIKE CLASS**	**TXL1**	**TXL2**	**TXL3**	**TXL4**	**OUTPUT (1st run)**	**OUTPUT (2nd RUN)**
**Ideal**	3H	0.7757	0.7657	0.7546	0.7443	0.03	0.06
**Rounded**		0.78	0.77	0.76	0.74		
**Ideal**	3M	0.7592	0.7510	0.7427	0.7350	0.08	0.16
**Rounded**		0.76	0.75	0.74	0.74		
**Ideal**	3L	0.7450	0.7397	0.7329	0.7266	0.25	0.39
**Rounded**		0.75	0.74	0.73	0.73		
**Ideal**	2H	0.7400	0.7270	0.7163	0.7071	1.00	1.19
**Rounded**		0.74	0.73	0.72	0.71		
**Ideal**	2M	0.7347	0.7231	0.7149	0.7094	0.99	1.23
**Rounded**		0.73	0.72	0.71	0.71		
**Ideal**	2L	0.7164	0.7058	0.6983	0.6923	0.58	0.83
**Rounded**		0.72	0.71	0.70	0.69		
**Ideal**	1H	0.7151	0.7055	0.6971	0.6904		
**Rounded**		0.72	0.71	0.70	0.69		
**Ideal**	1M	0.7115	0.7014	0.6926	0.6863	0.36	0.62
**Rounded**		0.71	0.70	0.69	0.69		
**Ideal**	1L	0.6991	0.6888	0.6788	0.6705	0.14	0.28
**Rounded**		0.70	0.69	0.68	0.67		

Supplementary table 3: Results for texel array experiment. Ideal (computed) and rounded values used as voltage inputs to the texel array elements (TXL1-4) are shown alongside the resulting output voltages at node VOUT for two repetitions of the experiment. All units are Volts.

	**TXL1**	**TXL2**	**TXL3**	**TXL4**
**Before run**	19.5k	14.8k	12.7k	10.6k
**After run**	19.5k	15.1k	12.6k	10.6k

Supplementary table 4: Resistive states of memristors used for texel array experiment as measured before and after experimental run.

Supplementary notes:

Supplementary note 1 – Generalised approach to designing analogue gates using memristors

The fundamental principle behind our proposed paradigm is illustrated in its most general form in Supplementary Figure 9 for both the inverter and the NAND gate. Each memristor performs a unique function within each topology; modulating drain-source resistance or source-degenerating a transistor or both. Inverter case: R_A and R_D source-degenerate transistors M2 and M1 respectively whilst R_B and R_C modulate their effective drain-source resistances respectively. NAND case: similar to the inverter but R_F simultaneously source-degenerates M2 and modules effective drain-source for M1. It is interesting to note that the full general analogue inverter has therefore 4 degrees of freedom, whilst the fully general analogue NAND features 7 dofs; one less than twice the inverter's dofs. This is due to the shared functionality of R_F in the NAND gate. We therefore conclude that gates that include many instances where one transistor's source connects directly to exactly one transistor's drain will feature relatively fewer degrees of freedom. If, however, the transistors are not connected one source-to-one drain a memristor may be introduced in front of each transistor much like the configuration at the output of the NAND gate (node V_{OUT} in Figure 3a and Supplementary Figure 9).

Note: Interestingly, there is no reason why analogue gates cannot be implemented using transistor sizing techniques instead of memristors, provided all of the following conditions are met: i) The desired analogue gate transfer characteristic is known in advance, ii), it is possible to build the system within specs without requiring post-fab trimming and iii) reconfigurability is not required.

Supplementary note 2 – Programming the memristors

Reconfigurability in our circuits necessarily involves some overhead in the form of a programming structure. An example would consist of four practically minimum size transistors and is illustrated in Supplementary Figure 10 for the basic circuit in Figure 1. Its operation is based on a succession of program and assess phases, whereby the system applies a programming pulse to the device to be programmed and then assesses the new resistive state of the device, continuing to do so until the device resistive state matches the requirements.

We remark that depending on what is connected to the input and output of the inverter the circuit in Supplementary Figure 10a may be amenable to further simplification, e.g. by removing M_b and M_c or M_a and M_d (as might be done in the case where a number of these inverters are cascaded). Whilst the design proposed here is a starting point in practice simple programming structures are expected to be designed once the input and output connectivity of each analogue gate is defined. Furthermore, the fundamental concept behind this example circuit can be generalised to higher level gates: to each input and output node there will correspond a node isolation transistor (similar to M_c, M_d in Supplementary Figure 10a) and a voltage forcing transistor (similar to M_a, M_b) connecting the input/output node to an appropriate voltage level.

Let us consider our spike-sorting application example where the inverter forming the core of Supplementary Fig. 10a directly outputs to the input of another inverter. When the texel memristors

are being programmed the output inverter is parked. Therefore there is no need for transistor Md. Moreover, the circuit in Supplementary Fig. 10 does not control the memristive devices as and of itself. Rather it provides a route from the memristor terminals to the programming circuit and the power supplies and once a memristive device is subjected to appropriate voltage it will switch.

In the specific example of Supplementary Figure 10a: during the program phase, V_{CTRL} goes high connecting LINE1 to the input node and LINE2 to the output node of the inverter while isolating these nodes from all circuitry before and after the gate. LINE1 is the tasked with selecting which device is to be programmed. If LINE1 imposes a high voltage on the input node, then M1 activates the connection to GND and M2 remains shut, therefore selecting R_C for programming. Subsequently LINE2 can apply either a high programming voltage (perhaps at or higher than the full power supply depending on CMOS and memristor technology specifics) in order to program the memristor in one direction (e.g. towards lower resistance) or a low programming voltage, well below GND, in order to program the memristor in the opposite direction. The exact same procedure is applied symmetrically to program R_B. The necessary voltage for programming the states of memristors can be supplied by a time-shared pair of charge pumps located at the chip's periphery and only being powered up when programming is required. The charge pumps can fill a capacitor that holds enough charge to program the memristors, which typically require pJ-level programming energies[1]. The ability of our proposed circuit to program memristive devices is illustrated through simulations in Supplementary Figure 10b.

In the assessment phase the proposed structure allows a number of possible options. In the simple case LINE1 remains a digital signal and selects a memristor to be tested as before, and then LINE2 provides a metered current (e.g. through a Transimpedance Amplifier) to the device and assesses its resistive state. A look-up table-based system can then determine target values for the memristor as appropriate and utilise the traditional Incremental Step Pulse Programming protocol as used in flash memory[2] to lead the devices to the correct state[3]. Another option is to use LINE1 as an analogue signal and then sweep the input voltage of the inverter in order to assess its full transfer characteristic. We note that as this system is designed for programming a few times and reading many, it is possible to afford chip-level shared programming systems that are even more elaborate than the minimum examples we provide here to support this work.

Importantly, this system requires only three signal lines to leave each texel: V_{CTRL}, LINE1 and LINE2. These lines may run without loss of generality in the vertical direction, i.e. be shared across an entire column of texels, in which case adding another minimum size transistor connected to an ENABLE line running in the horizontal direction (shared across rows) allows the addressing of texels in a cross-bar fashion. A number of tweaks to this circuit may be possible and improve performance further, but the fundamental principle remains that the long lines spanning the texel array column- and row-wise carry signals only whilst the device states are being modified. In normal operation M_a and M_b remain open whilst M_c and M_d are closed, thus allowing all signals to remain local whilst adding some parasitic capacitance at the input and output nodes of the inverter.

Finally we note that using memristors as opposed to flash introduces a number of potential advantages: i) Flash memory requires voltages in the range of 10V and above for successful programming whilst our memristive devices only require in the order of 1-2V typically for switching their resistive states. ii) The memristive devices are fabricated in the back-end-of-line and do not compete with transistors for silicon real estate. Furthermore, they can be downscaled to sizes

comparable with the gate length (not the full transistor size) of very advanced CMOS nodes[4]. iii) More speculatively perhaps, there is intense research in different memristor technologies that feature different operating voltages and resistive state ranges amongst other features. It is conceivable that in the future a small zoo of possible memristive device technology flavours might be available for designers, just as different flavours of transistors (e.g. different gate oxide thicknesses and doping specs) often exist in process development kits (PDKs). This might lead to considerable additional design flexibility, not available currently in CMOS despite the different transistor options.

Supplementary note 3 - Estimating power dissipation

A crucial aspect of the proposed design is its power efficiency. This is best illustrated by inspecting the energy dissipation of an ideal digital inverter and its analogue counterpart. The energy required to flip a standard inverter's state E_{flip} depends on the output capacitance C_{out} and the power supply voltage VDD and is given by the well-known formula:

$$E_{\text{flip}} = C_{\text{out}} \frac{VDD^2}{2} \tag{1}$$

In order to investigate power dissipation in an analogue, reconfigurable inverter we consider a very simplified circuit where both transistors and memristors are treated as linear resistors that remain constant for any fixed input V_{IN} as illustrated in Supplementary Figure 2b. The objective is to compute the energy cost involved in moving the output voltage of the analogue inverter from its initial state $V_{\text{OUT,1}}$ under input voltage $V_{\text{IN,1}}$ to a new state $V_{\text{OUT,2}}$, as imposed by a new input voltage $V_{\text{IN,2}}$. The current leaving the power supply I_{VDD} is given by Kirchhoff's law:

$$I_{\text{VDD}}(t) = \frac{\text{VDD} - V_{\text{OUT}}(t)}{R_1} = \frac{\text{VDD} - \Delta V_{\text{OUT}} e^{-t/R_{||}C_{\text{out}}} - V_{\text{OUT,2}}}{R_1} = \frac{\text{VDD}(1 - Q_{\text{div}}) - \Delta V_{\text{OUT}} e^{-t/R_{||}C_{\text{out}}}}{R_1} \tag{2}$$

where $Q_{\text{div}} = \frac{R_2}{R_1 + R_2}$, $\Delta V_{\text{OUT}} = V_{\text{OUT,2}} - V_{\text{OUT,1}}$, $R_{||} = \frac{R_1 R_2}{R_1 + R_2}$, R_1, R_2 the equivalent memristor-transistor resistances at $V_{\text{IN}} = V_{\text{IN,2}}$ and keeping in mind that $V_{\text{OUT,2}} = \text{VDD} \cdot Q_{\text{div}}$.

Integrating (2) over time for an interval of time $t_{\text{set}} \equiv l R_{||} C_{\text{out}}$ where we consider the system to have satisfactorily converged to its equilibrium value ($V_{\text{OUT,final}} \approx V_{\text{OUT,2}}$) we obtain total charge usage Q_{tot} of:

$$Q_{\text{tot}}(t_{\text{set}}) = \int_{t=0}^{t=t_{\text{set}}} I_{\text{VDD}} dt = t_{\text{set}} \frac{\text{VDD}}{R_1 + R_2} + C_{\text{out}} \Delta V_{\text{OUT}} Q_{\text{div}} (1 - e^{l}) \tag{3}$$

We notice that the first term is a constant leakage down the inverter (leakage term) and it depends on the total inverter impedance and the time necessary for the computation to be concluded to within tolerance. This is illustrated in Supplementary Figure 2b as i_{leak}. The second term includes the ideal charge transfer required to change the voltage at the output node by ΔV_{OUT}, $Q_{\text{ideal}} = C_{\text{out}} \Delta V_{\text{OUT}}$ (charging term i_{cap} in Supplementary Figure 2b). The Q_{div} in the charging term is best understood as the extent to which the C_{out} capacitor current flows into the power supply or the ground. With Q_{div} close to zero C_{out} charges/discharges preferentially into the ground (similar to a standard inverter toggling from output 1 to 0 – the output signal transition is achieved primarily by

sinking charge from the output capacitance into ground) whilst for Q_{div} close to unity the supply is preferred (standard inverter toggling from output 0 to 1). We also note that the charging term may be positive or negative depending on the relationship between $V_{\mathrm{OUT},2}$ and $V_{\mathrm{OUT},1}$. Without loss of generality we consider the case where $V_{\mathrm{IN},1} > V_{\mathrm{IN},2}$, $V_{\mathrm{OUT},1} < V_{\mathrm{OUT},2}$ and the charging term is positive.

Translating charge into energy dissipation we can compute an upper bound by rounding Q_{div} to unity and considering that every charge q leaving the power supply will (eventually) reach ground dissipating $q\mathrm{VDD}$ energy. We thus obtain:

$$E(t) < Q_{\mathrm{tot}}(t_{\mathrm{set}})\mathrm{VDD} < \; t_{\mathrm{set}}\frac{\mathrm{VDD}^2}{R_1+R_2} + \mathrm{VDD}C_{\mathrm{out}}\Delta V_{\mathrm{OUT}}(1-e^{l}) \tag{4}$$

This highlights three points: First, the analogue inverter has an (upper bound) energy dissipation given by a charging term that reduces to the standard inverter (within factor of 2) dissipation for $\Delta V_{\mathrm{OUT}} = \mathrm{VDD}$ and $t \rightarrow \infty$ plus a leakage term. Second, the leakage term depends on the in-operando impedance of the inverter whilst the charging term only depends on output capacitance. Third, longer waiting times lead to more accurate computations ($V_{\mathrm{OUT}}(t_{\mathrm{set}})$ closer to the ideal equilibrium value), but incur a larger leakage energy penalty.

Finally, it can be shown that by expressing $R_1 + R_2$ in terms of Q_{div} and t_{set} in terms of l, eq. (3) can also be expressed as:

$$Q_{\mathrm{tot}}(t_{\mathrm{set}}) \; = \; lC_{\mathrm{out}}\mathrm{VDD}Q_{\mathrm{div}}(1-Q_{\mathrm{div}}) + C_{\mathrm{out}}\Delta V_{\mathrm{OUT}}Q_{\mathrm{div}}(1-e^{l}) \tag{5}$$

The fact that charge consumption can be expressed as function of Q_{div} only, shows that the absolute values of R_1 and R_2 are only significant for setting the temporal dynamics (via the $R_{||}C_{\mathrm{out}}$ constant – the units of l); charge dissipation depends only on their relationship. This is significant because it suggests that analogue gate speed can be traded off against memristor resistive state. Eq. (5) also reveals that for $\Delta V_{\mathrm{OUT}} \approx \mathrm{VDD}$ and suitable Q_{div} the leakage and charging terms will be broadly comparable even for l values sufficiently long to allow the system to converge to equilibrium (e.g. within 2% for $l = 4$).

In conclusion, the calculations above suggest that analogue computation is achievable at an energy price close to digital under the simplifying assumptions about transistor and memristor resistances made by equations (1-5). Moreover, in practical electronics input voltages can only cross from one level to the other within a finite interval of time. This causes even the standard Boolean inverter to spend some time with both its transistors ON when the input voltage is between digital 1 and 0 with an associated energy cost ignored by eq. (1). Mathematically, this is tantamount to noting that purely digital gates too suffer from the leakage term in supplementary eq. (5), even if to a lower extent. The precise impact of finite input transition times on the results in eq. (4) lies outside the scope of this paper. Results on power dissipation are in line with expectations from the simulations on more realistic memristor-enhanced and standard inverters, implemented in a 0.35 micron commercially available technology that are shown in Supplementary note 4.

Supplementary note 4 - Power estimations for analogue inverter operation and benchmarking

Estimation of the operating power budget of an analogue inverter was investigated through simulation on the industry-standard Cadence tool (see Supplementary methods). In order to give a very conservative and operationally relevant estimate, a modified version of a full texel circuit, as illustrated in Figure 4a, was simulated (full texel schematic used in Supplementary Figure 11b) within the power dissipation estimation test bench shown in Supplementary Figure 11a. The reference technology was a commercially available CMOS technology (AMS 0.35 micron, C35). Resistors were used to model memristors, and the amount of charge removed from the power supply to carry out the computation was taken as a proxy for power dissipation.

Operating power estimations were benchmarked for the following analogue computation: input voltage rises from 1.55V to 1.7V. These values guaranteed a visible change in the system output voltage level, as evidenced in Supplementary Figure 12. By the time the output voltage stabilises the overall amount of charge removed from the power supply is approximately 46fC. This compares favourably with the ~1.25fC charge dissipated by an industrially-designed minimum size inverter for a single digital state transition (input 0 to 1) in the same technology, as shown in Supplementary Figure 13. Therefore for the charge dissipation price of ~39 inverter toggles the texel carries out an analogue input-output mapping operation. Notably, the energy price includes the operation of two analogue inverters (driving inverter from test bench and inverter included within texel) plus the read-out stage. Furthermore, the texel circuit used in this study was not optimised for low power dissipation but is provided as a working example that can be set up with minimum design effort.

The major competing approach would be to perform the same computation using a digital range comparator capable of telling whether some input value x lies within some interval [V_{low}, V_{high}]. Such range comparator can be constructed out of two simple comparators similar to the 4-bit design we simulated for this work and which is shown in Supplementary Figure 14. In technology C35 under 3.3V power supply and using industrially designed gates, the cost of a half-comparison was 150fC, which is already significantly more than the dissipation of the texel for a full range comparison. Notably the 150fC figure was obtained for an LSB's worth of change in the digital input of the range comparator. Importantly we note that the digital comparator maintains full flexibility of the comparison limits, which span the entire input space range. Thus, the texel and the standard digital comparator are complementary in that they operate at different points of the raw energy consumption/operational flexibility design space. Furthermore, we note that the very same computation carried out in anything more complicated than the minimum gate example above (GPU, CPU, FPGA) would likely carry a significantly higher energetic cost (though a thorough energetic study of a look-up table (LUT)-based implementation is recommended – outside the scope of the current paper). As a result, there are good reasons to expect that the memristor-based approach explained in this paper has a chance of obtaining a competitive advantage over traditional implementations in at least some tasks where the constraints and requirements match what the approach has to offer.

Finally, comparing the proposed circuit against the 4-bit range comparator approach we note that the texel design consists of far fewer, albeit large transistors. This directly evidences the increased computational functionality of the texel vs. purely digital approaches. Furthermore, observing Supplementary Figure 11b we notice that the combined transistor gate area in the texel circuit (inverter + read-out) is 26x0.35μm^2, i.e. equivalent to 74 minimum size transistors (0.35x0.35μm^2). By comparison, the range comparator has an overall gate area of approx. 77.6 μm^2. However: first we note that this is not yet an optimised design. Second the smaller number of transistors (6 vs 52) means that overheads such as minimum spacing rules and area reserved for the source and drain terminals (which do not scale well at all as the technology pushes towards the 7nm and 5 nm nodes)

are kept under control. As an example let us consider that a single, industrially designed minimum size inverter occupies $38\mu m^2$ of area even though the area under gate is only $0.45\mu m^2$. Third, the ability of memristors to act as trimming elements allows the design of analogue circuits with a fraction of the additional area used previously for the purposes of combating process variation.

Supplementary note 5 – Operational device programming

In order to program memristive devices an automated algorithm is used. This builds on the incremental step pulse programming approach used in flash memory[2] and our own previous work concerning how to efficiently drive memristive devices across their resistive state range[3]. We demonstrate the degree to which we can control our devices in supplementary figure 15 using three examples. The test devices were cycled through a schedule passing through the resistive states [25kΩ - 40kΩ - 32kΩ - 31kΩ]. The programming error tolerance was 1% of the nominal resistive state target value.

Results indicate that it is possible to set device resistive states accurately (well within the 1% tolerance limit set for these tests in most cases) using largely automated methods even in our university cleanroom technology. The example of device under test (DUT) 3 illustrates that sometimes devices may show signs of weak volatility and require a second round of coaxing in order to reach the target resistive state. This occurs just before DUT3 reaches the 24.96kΩ and 39.80kΩ marks. The algorithm was then simply reran and stable convergence was then achieved. We offer the conclusion that checking for volatility and rerunning the algorithm is easy to automate and may be a good checking step when programming memristive devices in the analogue domain automatically. Volatility/drift effects were checked for by taking a few reads manually between the runs of the automatic algorithm. These can be discerned in supplementary figure 15 as blocks of pulses at the 0.2V level. More information on the retention capabilities of our devices can be found in the literature[1].

Supplementary note 6 – Additional information of interest

This section briefly covers a collection of points that are deemed to be of general interest and not expressly covered in other sections:

Timing is important: The leakage term in supplementary eq. (5) is tightly linked to time, through variable l, which encodes the time spent settling. Whilst the full details of an architecture (or at least medium complexity system module) that operates on the basis of the proposed analogue gates are still being worked out, it is immediately clear that appropriate clocking control will be necessary. In a nutshell, we do not want our system to remain leaking unless we are actually computing something (in analogue). This leads to an interesting observation: If we are constantly making computations and inputs keep arriving at a steady and appropriate pace, then the leakage and charging terms always remain similar in magnitude, therefore we are continuously operating individual analogue gates at a price slightly higher than operating digital gates.

Parking analogue gates: If for whatever reason we wish to stop computing using these analogue gates we can very easily park them in full-digital mode. This involves feeding them clean digital

signals and then letting them behave (almost) exactly as standard digital gates, where the transistors are never simultaneously on. This ability to park gates is seen as an important feature of future designs using analogue gates.

On the potential to improve power dissipation figures: In supplementary figure 12 it is shown how the system described in supplementary figure 11 dissipates power as it performs an example analogue computation. We note that much of the power is dissipated waiting for the final output to converge. Optimising the design of the two inverters shown in supplementary figure 11b in order to harmonise their dynamics (i.e. making sure that the answer of the 2nd stage is readily available immediately after the answer from the 1st stage) is expected to have a major impact on dissipation. Another possible way (less preferable) would be separating the clocking for the 2 stages so that the faster, first stage analogue inverter is parked while the output stage inverter is still computing. This seems to indicate that there is plenty of room at the bottom.

Finer effects of introducing memristors in a logic gate: As briefly covered in supplementary note 3, even in digital gates there is a portion of time as they switch when both transistors are simultaneously on. In that very brief instant there is a peak in power dissipation when the DC impedance from VDD to GND is approximately given by the series resistances of the two transistors in the inverter. That peak can be quite sharp, but the memristive elements introduce a de facto current-limiting resistance. This can act as a damper and limit the size of the current spikes during switching (with effects on noise, not just power dissipation – potentially useful in mixed signal circuits, where digital noise affecting the analogue part is highly problematic).

On the possibility of obtaining a larger sensed voltage range from our template matcher application: The precise range of sensed voltages we can obtain will strongly depend on the range of resistances that the memristors employed can cover. SPICE simulations on the circuit shown in Figure 2b indicate that manipulating memristive device resistances only it is possible to widen the input range to approximately 0.5V out of a power supply VDD=1.65V. This is shown in supplementary figure 16. As a side note, devices covering similar ranges have already been reported in the literature e.g. by NIST[5] and memristor technology continues advancing at very rapid rates. Moreover, other methods for achieving higher input voltage ranges also exist, including tuning the output stage inverter as shown in supplementary figure 11b or using the full flexibility of the design shown in supplementary figure 9a. In summary, there seems to be plenty of room at the bottom.

Supplementary methods:

All experimental work carried out for the Supplementary material followed the same basic procedures and used the same instrumentation as explained in the methods section of the main text. All proof-of-concept level work e.g. Supplementary figures 3, 4, 6, 10 and 16, was carried out using TSMC's MOSIS 0.35 micron technology with a power supply of 1.65V unless otherwise stated and LTSPICE. Note: 0.35 micron technology operates at 3.3V typically. However, for these experiments we lowered the supply to 1.65V, which is another instance of power supply vs. memristive device resistive state tailoring. We further observe that: First, this system can coexist as a module within a standard 0.35 micron technology where the rest of the system operates at the 3.3.V supply. Second, as we apply the same design approach to smaller and smaller nodes we can expect there to be a sweet spot where the power supply leads to striking the right balance between memristor and transistor resistances. Finding this merits its own, dedicated analysis. All quantitative analysis work, e.g. supplementary figures 12 and 13, was carried out using AMS' 0.35 C35 micron technology under

a 3.3V power supply using Cadence. Component sizings are shown on the schematic of Supplementary Figure 11b. The read-out stage of the texel circuit in Supplementary Figure 11 was itself enhanced with memristors in order to drop overall power dissipation.

The tests ran for supplementary note 5 and supplementary figure 15 were carried out on the ArC ONE platform using pulsed voltage train ramps with the following parameters: 15 pulses per voltage level used before proceeding to the next increment; 0.1V voltage step; inter-pulse interval of 10ms; pulse duration 1μs. Minimum attempted pulsed voltage was varied manually to save time.

Supplementary references:

1. S. Stathopoulos, A. Khiat, M. Trapatseli, S. Cortese, A. Serb, I. Valov, and T. Prodromakis, "Multibit memory operation of metal-oxide bi-layer memristors," *Sci. Rep.*, vol. 7, no. 1, p. 17532, Dec. 2017.

2. Kang-Deog Suh *et al.* A 3.3 V 32 Mb NAND flash memory with incremental step pulse programming scheme. *IEEE J. Solid-State Circuits* **30,** 1149–1156 (1995).

3. A. Serb, A. Khiat, and T. Prodromakis, "A biasing parameter optimiser for RRAM technologies," *IEEE Transactions on Electron Devices*. 29-Jun-2015.

4. Khiat, A. *et al.* High Density Crossbar Arrays with Sub- 15 nm Single Cells via Liftoff Process Only. *Sci. Rep.* **6,** 32614 (2016).

5. N. Gergel-Hackett, B. Hamadani, B. Dunlap, J. Suehle, C. Richter, C. Hacker, and D. Gundlach, "A flexible solution-processed memristor," *IEEE Electron Device Lett.*, vol. 30, no. 7, pp. 706–708, Jul. 2009.
